# Twenty-Year Trends in Malaria Epidemiology, Importation Dynamics, and Surveillance in Oman: A Retrospective National Surveillance Study

**DOI:** 10.3390/pathogens15101036

**Published:** 2026-10-01

**Authors:** Mawahib Ahmed, Basmah Alharbi, Omar Al Farsi, Raniya Al Kiyumi

**Affiliations:** 1 Department of Medical Laboratories, College of Applied Medical Sciences, Qassim University, Buraidah 51452, Saudi Arabia; 2 Department of Basic Health Sciences, College of Applied Medical Sciences, Qassim University, Buraidah 51452, Saudi Arabia; b.alwahbi@qu.edu.sa; 3 Health Information Management Program, Oman College of Health Sciences, Muscat 112, Oman; omar.alfarsi@ochs.edu.om

**Keywords:** imported malaria, malaria elimination, epidemiology, *Plasmodium* species, retrospective observational study, Oman

## Abstract

Background: Malaria remains a major vector-borne disease worldwide. Oman interrupted endemic malaria transmission in 2010, but imported infections and occasional locally acquired cases continue to require sustained surveillance. This study evaluated national malaria epidemiology and surveillance patterns across the complete 2005–2024 period. Materials and Methods: We conducted a retrospective observational analysis of aggregated malaria surveillance indicators reported by the Oman Ministry of Health and linked national surveillance sources. The analytical series comprised 20 consecutive annual observations (2005–2024). Indicators included Annual Parasite Incidence (API), Slide Positivity Rate (SPR), Annual Blood Examination Rate (ABER), blood slides examined, case origin, surveillance approach, *Plasmodium* species distribution, and 2024 governorate-level case counts. Descriptive analyses, linear temporal models, robust Poisson rate models, regression diagnostics with Newey–West sensitivity analyses, and a 2024 population-referenced geographic analysis were performed. Results: Across all 20 years, 17,080 malaria cases were reported; 16,905 (98.98%) were imported, 174 (1.02%) were autochthonous, and one case was unclassified by origin. API declined from 0.217 per 1000 population in 2005 to 0.064 in 2024. ABER and annual blood-slide examination volume declined, whereas SPR increased over time. The temporal slope for blood slides examined was strongly negative (standardized β = −0.907, *p* < 0.001). The annual testing-volume–SPR association was inverse and statistically significant in the restored 20-year series (B = −0.00000147, 95% CI −0.00000254 to −0.00000040, *p* = 0.010; R^2^ = 0.317); the association remained significant with Newey–West lag-1 standard errors (*p* = 0.019). Among the two principal species, the relative annual rate of *P. falciparum* increased (aRR = 1.142, 95% CI 1.068–1.221, *p* < 0.001), whereas that of *P. vivax* decreased (aRR = 0.952, 95% CI 0.919–0.987, *p* = 0.007). In 2024, crude reported case rates varied across governorates, but these rates combine imported and locally acquired cases and therefore do not represent local transmission incidence. Conclusion: Surveillance during 2005–2024 was characterized by persistent predominance of imported malaria, sparse autochthonous cases, changing species composition, and declining testing-volume indicators. The findings support continued diagnostic readiness and surveillance for imported infection and possible re-establishment, without implying effectiveness of any specific intervention.

## 1. Introduction

Malaria remains a major global public-health challenge despite substantial progress in control and elimination [1]. The continuing movement of infected travellers and migrants means that countries with interrupted endemic transmission can still diagnose imported malaria and remain at risk of re-establishment when competent vectors and receptive conditions are present [2,3]. Consequently, elimination settings require surveillance systems that can rapidly detect, classify, investigate, and manage imported and locally acquired infections [4].

Oman launched its national malaria eradication programme in 1991 and subsequently achieved a marked reduction in indigenous transmission. The World Health Organization reported interruption of endemic malaria transmission in 2010; this status does not mean that all malaria diagnoses ceased, because imported infections have continued to be detected [5,6]. The national programme combines vector control, early case detection, prompt treatment, and intense treatment (which includes prompt diagnosis and radical curative treatment with artemisinin-based combination therapy followed by primaquine to clear sequestered blood-stage parasites and eliminate hypnozoites, thereby interrupting parasite transmission chains).

Case detection includes passive detection through routine presentation to health facilities and active approaches such as epidemiological contact surveys, targeted community screening, school screening, and screening of selected travellers or residents from malaria-endemic areas [7,8,9].

Laboratory quality assurance is an integral component of this surveillance system. Reported quality-control procedures in Oman include monthly cross-checking at regional and central laboratories of all positive slides and a sample of negative slides, immediate review of doubtful results, periodic supervisory visits to facilities providing malaria microscopy, and training or refresher activities for laboratory personnel [10]. These procedures are intended to support reliable species identification and parasite detection and are not an intervention outcome evaluated in the present study.

Microscopy of thick and thin blood films remains central to malaria diagnosis, with rapid diagnostic tests and molecular methods used in selected circumstances [11]. In Oman, once a test is positive, clinicians aim to identify the species and to begin the required treatment, which the state provides free of charge, on the same day. The case is then immediately reported to the disease surveillance system [12].

In an elimination setting, the epidemiological classification of every confirmed infection is also important because imported and autochthonous cases have different implications for re-establishment risk [13]. Oman nevertheless faces continuing surveillance challenges, including imported infections, occasional local transmission events, changing *Plasmodium* species patterns, and the need to maintain diagnostic capacity when malaria burden is low [14].

The present study therefore examined long-term changes in malaria burden and surveillance indicators across the 2005–2024 period using the annual observations available in the compiled national dataset. Specifically, it evaluated Annual Parasite Incidence (API), Slide Positivity Rate (SPR), Annual Blood Examination Rate (ABER) testing volume, case origin, *Plasmodium* species composition, and passive versus active testing. Oman comprises 11 governorates; because a complete longitudinal set of governorate-specific denominators was not available for the full study period, geographic comparison was restricted to 2024, for which reported case counts were contextualized using end-2024 registered governorate populations from the National Centre for Statistics and Information. This approach provides a population-referenced descriptive comparison while avoiding unsupported inferences about the causes of geographic differences.

## 2. Materials & Methods

### 2.1. Study Design

This retrospective observational study analysed aggregated malaria surveillance information from the Oman Ministry of Health annual health-report series and related national surveillance publications [15]. The study window and analytical series both spanned 20 consecutive calendar years from 2005 through 2024. The 2006–2009 observations were restored from the original 20-year source data rather than treated as missing. For these years, national totals for blood slides, positive cases, and species were taken from the original annual records; API and ABER were calculated using the same definitions and annual population denominators applied across the series, and case-origin classifications were restored from national surveillance reporting. The calendar year was the unit of analysis for national temporal analyses. The indicators were API (confirmed malaria cases per 1000 population under surveillance), SPR (malaria-positive blood slides divided by all blood slides examined × 100), ABER (blood slides examined divided by the population under surveillance × 100), total blood slides examined, total reported cases, case origin (imported, autochthonous, or unclassified where applicable), *Plasmodium* species, and passive/active testing counts. Imported cases were those classified by the surveillance source as acquired outside Oman; autochthonous cases were classified as locally acquired. Passive testing represented testing initiated through routine presentation to health facilities, whereas active testing represented targeted screening and surveillance activities. These definitions follow the national reporting framework and WHO malaria-surveillance terminology [16]. The Ministry of Health Annual Health Reports page was used as the report-series reference. For the 2024 geographic comparison, end-2024 registered populations for all 11 governorates were obtained from the Oman National Centre for Statistics and Information Statistical Year Book 2025 [17]. Monthly data, complete year-by-governorate denominators for the full period, individual-level records, and species-by-origin cross-tabulations were not consistently available and were not reconstructed or imputed.

### 2.2. Statistical Analysis Plan

Descriptive statistics summarized each calendar year using counts, percentages, and reported or consistently derived rates. Linear temporal models used calendar year as a continuous predictor for API, SPR, ABER, and blood slides examined (*n* = 20 annual observations for each model). The unstandardized coefficient (B) therefore represents average annual change rather than comparison with a reference year. The association between annual testing volume and SPR was examined by simple linear regression (*n* = 20), with SPR as the outcome and blood slides examined as the predictor. For each linear model, residual normality, variance pattern, and residual dependence were evaluated using Q–Q plots/Shapiro–Wilk tests, residual-versus-fitted plots/Breusch–Pagan tests, and Durbin–Watson statistics, respectively. Heteroscedasticity- and autocorrelation-consistent Newey–West (lag 1) standard errors were used as a sensitivity analysis because residual dependence could not be excluded for some annual series.

Case-origin and species comparisons were analysed using log-link Poisson generalized linear models rather than logistic odds models. For each analysis, the data were placed in long format with two outcome categories observed in each of the 20 calendar years. Thus, the term “40 category-year observations” refers to 20 years × 2 categories; calendar year was not treated as a 40-level categorical variable. The models included category, centered calendar year, and the category × year interaction, with the log of the relevant annual classified total as an offset and robust sandwich standard errors. The interaction term directly tested whether the temporal rate of change differed between the two categories. For case origin, 40 category-year observations (imported and autochthonous across 20 years) were analysed; one unclassified-origin case in 2022 was retained in descriptive totals but excluded from the two-category rate model. For species, 40 category-year observations representing *P. vivax* and *P. falciparum* across 20 years were analysed; infections attributed to other or unclassified species were not included in that two-species model. Exponentiated coefficients are reported as adjusted rate ratios (aRRs) with 95% confidence intervals. Passive/active testing was analysed descriptively because the available values represent slides tested rather than confirmed malaria-positive cases by detection approach; approach-specific counts for 2006–2009 were not available in the compiled workbook, although those years remained included in all analyses for which the relevant national indicators were available.

The 2024 geographic analysis included all 11 governorates (*n* = 11) and calculated a crude reported malaria case rate per 100,000 registered population as follows: reported 2024 malaria cases ÷ end-2024 registered population × 100,000. This is a descriptive population-referenced case rate, not a measure of locally acquired transmission. The available governorate cross-tabulation contained total reported malaria cases but did not separately identify imported and autochthonous cases by governorate. Accordingly, a governorate-level correlation between imported and autochthonous cases could not be calculated without reconstructing or imputing data that were not present in the source dataset. Joinpoint analysis was re-evaluated after restoration of all 20 annual observations. Although the longer series resolves the prior observation-count concern, autochthonous counts remain sparse and include multiple zero-count years. Alternative segmented specifications were sensitive to the treatment of zero counts and produced unstable change-point locations; therefore, Joinpoint was not used for primary inferential conclusions. The robust Poisson rate model across all 20 years was retained as the principal analysis of case-origin trends. For inferential models, two-sided t or Wald tests evaluated H0: coefficient = 0 at α = 0.05, with corresponding 95% confidence intervals. Analyses of aggregated surveillance data were interpreted as ecological temporal associations and not as individual-level or causal effects.

## 3. Results

Across the complete 20-year series (2005–2024), national surveillance showed a broad decline in API, ABER, and blood-slide examination volume, alongside an overall increase in SPR (Table 1). API fell from 0.217 per 1000 population in 2005 to 0.064 in 2024, while ABER declined from 10.32% to 0.67%. The highest API was 0.566 in 2012. The restored 2006–2009 observations show the expected transition between the 2005 and 2010 values rather than a four-year gap in the time series. The marked reduction in testing volume after 2019 coincided with higher SPR values in several later years, but this temporal co-occurrence does not establish causation.

**Figure 1:**
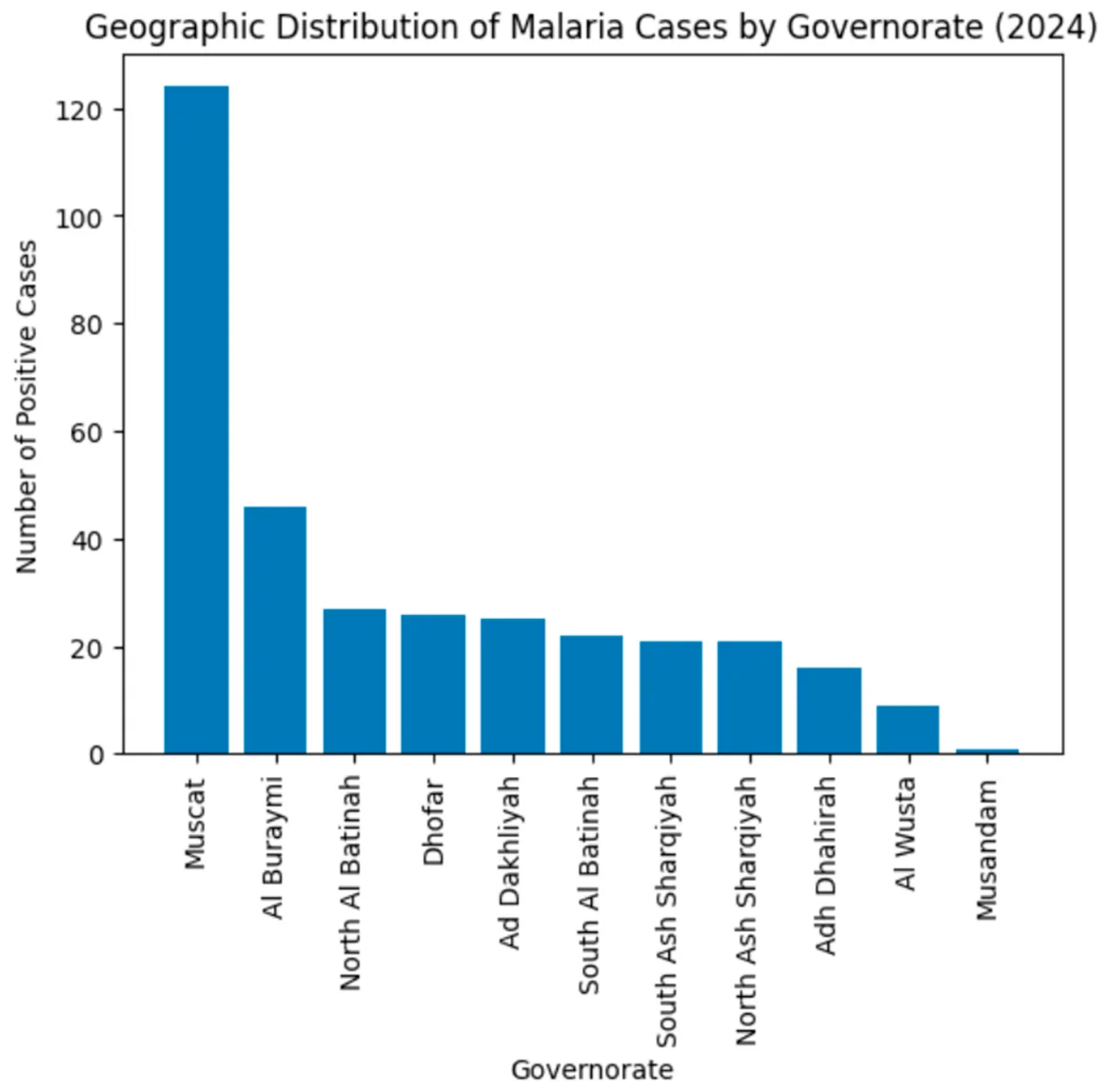
Reported malaria case counts by governorate, Oman, 2024. Note: Values are absolute reported case counts, not population-adjusted incidence.

Geographic comparison for 2024 showed substantial variation in both absolute counts and population-referenced crude rates (Figure 1 and Table 2). Muscat recorded the largest absolute number of cases (124), whereas Al Buraymi had the highest crude reported case rate (33.9 per 100,000), followed by Al Wusta (14.7 per 100,000) and Muscat (8.3 per 100,000). Musandam recorded one case (1.8 per 100,000). These values describe where cases were reported and should not be interpreted as governorate-specific local transmission incidence because case origin was not available within the geographic cross-tabulation.

In the restored 20-year dataset, annual blood-slide examination volume was significantly and inversely associated with SPR. The estimated coefficient was B = −0.00000147 per additional slide (95% CI −0.00000254 to −0.00000040, *p* = 0.010; R^2^ = 0.317; *n* = 20), corresponding to a standardized β = −0.563 (Table 3). The residual diagnostics did not indicate non-normality or heteroskedasticity (Shapiro–Wilk *p* = 0.378; Breusch–Pagan *p* = 0.860), although modest residual dependence remained possible (Durbin–Watson = 1.21). The association remained statistically significant using Newey–West lag-1 standard errors (*p* = 0.019). This ecological association does not establish that lower testing volume caused higher positivity.

Case-origin composition remained overwhelmingly imported throughout the complete series (Table 4). In the robust Poisson rate model using all 20 years, the annual relative rate of autochthonous cases did not show a statistically significant linear change (aRR = 1.048 per year, 95% CI 0.984–1.116, *p* = 0.148), and the imported-case rate was essentially stable relative to the classified annual total (aRR = 1.000, 95% CI 0.999–1.000, *p* = 0.170). The category-by-year interaction was also not significant (ratio of rate ratios = 1.048, 95% CI 0.984–1.116, *p* = 0.144). Thus, restoration of 2006–2009 did not support a simple monotonic increase or decrease in case-origin composition. These fluctuations are not interpreted as evidence of interruption, persistence, or resurgence of local transmission.

Species composition changed substantially over the 20-year period (Table 5). *P. vivax* predominated among the two principal species through 2017, whereas *P. falciparum* predominated from 2018 through 2021 before the relative balance shifted again in 2022–2024. In the robust Poisson rate model using all 20 years, the relative annual rate of *P. falciparum* increased (aRR = 1.142, 95% CI 1.068–1.221, *p* < 0.001), while that of *P. vivax* decreased (aRR = 0.952, 95% CI 0.919–0.987, *p* = 0.007). The species-by-year interaction was significant (*falciparum*-to-*vivax* ratio of rate ratios = 1.199, 95% CI 1.112–1.293, *p* < 0.001). These results describe species composition among the two principal species and cannot identify whether the shift arose within imported or locally acquired infections because species-by-origin cross-tabulations were unavailable.

The balance between passive and active testing also varied over time (Table 6). Approach-specific passive/active counts were available for 2005 and 2010–2024 in the compiled workbook; 2006–2009 are displayed as not reported rather than being removed from the study period. Active testing accounted for the larger recorded share of classified testing in several years from 2012 to 2019, while passive testing again predominated from 2020 onward. These are testing-volume data only; confirmed malaria-positive cases were not cross-tabulated by surveillance approach, so the table cannot be used to compare the effectiveness or diagnostic yield of passive versus active strategies.

The linear temporal models using all 20 annual observations confirmed significant downward trends in API, ABER, and blood slides examined and a significant upward trend in SPR (Table 7). API decreased by an estimated 0.0142 per 1000 population per year (β = −0.597, *p* = 0.005), SPR increased by 0.0283 percentage points per year (β = 0.732, *p* < 0.001), ABER decreased by 0.537 percentage points per year (β = −0.985, *p* < 0.001), and blood-slide volume decreased by approximately 13,425 slides per year (β = −0.907, *p* < 0.001). Residual normality was acceptable for all four models (Shapiro–Wilk *p* ≥ 0.369), and Breusch–Pagan tests did not indicate heteroskedasticity (all *p* ≥ 0.215). Durbin–Watson statistics ranged from 0.56 to 1.59, indicating possible positive residual dependence in some series. Newey–West lag-1 sensitivity analyses retained the same direction and statistical-significance conclusions for all four trend models (API *p* = 0.014; all other *p* < 0.001).

As a secondary sensitivity analysis, Joinpoint regression of the complete 20-year series identified one joinpoint for API (2012) and SPR (2010) and two joinpoints for ABER and blood slides examined (2018 and 2021) (Table 8). API increased by 13.74% per year during 2005–2012 (95% CI 3.44–25.08; *p* = 0.011) and then decreased by 17.96% per year during 2012–2024 (95% CI −22.06 to −13.64; *p* < 0.001). SPR increased by 22.98% per year during 2005–2010 (95% CI 12.10–34.92; *p* < 0.001), whereas the subsequent 2010–2024 increase was smaller and not statistically significant (APC = 2.62%, 95% CI −0.26 to 5.58; *p* = 0.072). ABER and blood-slide examination volume showed their steepest declines during 2018–2021; neither indicator showed a statistically significant APC after 2021. These segmented estimates are interpreted as changes in temporal slope and do not imply effects of specific surveillance or control interventions.

## 4. Discussion

The principal observed pattern was a decline in several national malaria-burden and surveillance-volume indicators across the available annual observations. API decreased from 0.217 per 1000 population in 2005 to 0.064 in 2024, and both ABER and blood-slide examination volume declined over time. These findings document temporal change but do not, by themselves, demonstrate the effectiveness of specific malaria-control activities. Oman’s broader elimination programme and surveillance infrastructure provide relevant context [6,12,18], but causal attribution would require intervention-specific or individual-level designs.

Imported malaria was the dominant origin category throughout the observed series, while autochthonous cases remained uncommon. The Poisson model did not detect a significant annual change in the relative rate of either origin category. This pattern is compatible with the continuing importance of importation in an elimination setting, as described in Oman and other malaria-free or near-elimination contexts [2,3,19]. Potential explanations involving labour migration, travel volume, or source-country epidemiology are plausible hypotheses, but the present aggregated dataset did not contain traveller-level or migration data and therefore cannot test those mechanisms [20].

The 2024 geographic analysis adds population context to the absolute case-count figure. Although Muscat had the largest number of reported cases, Al Buraymi had the highest crude reported case rate per 100,000 registered population. This distinction illustrates why absolute counts alone should not be interpreted as regional burden. The reviewer-requested correlation between imported and autochthonous cases by governorate could not be estimated because the source geographic table does not cross-classify case origin by governorate. The geographic analysis therefore remains descriptive and combines imported and locally acquired infections.

Several mechanisms could plausibly contribute to geographic variation, but they are hypotheses rather than findings of the present dataset. Imported cases may cluster in governorates with greater international travel, migrant-worker residence, or movement from malaria-endemic countries, while locally acquired cases may depend additionally on ecological receptivity, vector presence, exposure around an imported index case, and the intensity and timeliness of surveillance and response. Oman’s elimination literature and the documented locally acquired *P. vivax* outbreak among migrant workers illustrate the importance of importation and subsequent local exposure in receptive settings [6,9]. However, because the present aggregated data do not link case origin, travel history, vector indicators, or individual exposures to governorate, these explanations cannot be tested here and should not be interpreted as causal explanations of the observed 2024 geographic differences.

Autochthonous case counts remained sparse and included several zero-count years even after restoration of the complete 20-year series. The robust Poisson rate model did not detect a statistically significant linear annual change in the relative autochthonous rate. For this reason, Joinpoint regression was not applied to case-origin counts. The secondary Joinpoint analysis was restricted to the positive continuous national indicators (API, SPR, ABER, and blood slides examined), where it identified changes in temporal slope that are reported as sensitivity results rather than as evidence of interruption, persistence, resurgence, or intervention effects. The World Health Organization’s report that Oman interrupted endemic malaria transmission in 2010 is a historical surveillance classification and is not inferred from the temporal models in the present study [5].

A marked change in the relative composition of *P. vivax* and *P. falciparum* was also observed, with *P. falciparum* becoming predominant during 2018–2021 before *P. vivax* again represented the larger share in 2022–2024. The rate model confirmed opposing temporal directions for the two species. However, species-by-origin information was unavailable, so the analysis cannot determine whether this pattern reflects changes among imported cases, local cases, or both. Shifts in countries of infection or migrant/traveller composition are possible explanations reported in the wider literature, but they remain hypotheses rather than conclusions from this study [21]. Although malaria is no longer endemic in Oman, the mosquitoes that transmit it remain widespread. The risk of malaria transmission depends on the mosquitoes’ geographic distribution, ecology, and vector competence, as well as favourable environmental conditions. This means the country is still vulnerable to malaria transmission. If an imported case introduces *P. vivax* or *P. falciparum* into an area where these mosquitoes are present, a localized outbreak may occur [9].

The composition of surveillance testing changed over time, with active testing representing a larger share of recorded examinations during several pre-2020 years and passive testing predominating after 2020. Because the source data provide numbers of slides tested rather than numbers of positive cases detected by each approach, these changes should be interpreted as surveillance-volume patterns only. They do not establish that one approach was more effective than another. Similar active case-detection programmes have been described in other elimination settings [22], but direct effectiveness comparisons require detection yields, target-population denominators, and programme-level covariates.

From a surveillance perspective, the findings support continued attention to imported malaria, maintenance of diagnostic readiness, and species-specific monitoring. The observed decline in testing-volume indicators is particularly relevant in a low-transmission setting because diagnostic capacity must remain sufficient to identify imported infections and investigate possible local transmission. These are programmatic implications derived from the surveillance patterns; they should not be read as evidence that any particular intervention caused the observed national trends. Notably, no recent documented policy changes have altered the testing-volume indicators.

Several limitations should be considered. First, the analysis used aggregated annual surveillance reports and cannot assess individual-level risk factors or causal relationships. Second, although all 20 calendar years from 2005 through 2024 were restored for the principal national analyses, autochthonous counts remained sparse with multiple zero-count years, precluding a stable Joinpoint analysis of case-origin counts, and possible residual dependence remained in some linear series. Third, monthly data were unavailable, preventing seasonal analysis. Fourth, complete longitudinal governorate-specific denominators and origin-stratified geographic data were not available; the geographic analysis was therefore limited to crude 2024 rates using end-2024 registered populations. Fifth, species-by-origin cross-tabulations were unavailable, and the two-species rate model excluded other or unclassified species. Sixth, passive/active surveillance counts for 2006–2009 were not available in the compiled approach-specific table, although these years were retained in the other 20-year analyses. Finally, the ecological testing-volume–SPR association is vulnerable to changes in testing indications and case mix. Future studies using line-list data, travel history, complete population denominators, and linked geographic, species, origin, and detection-method variables could evaluate seasonality, spatial patterns, importation pathways, and determinants of re-establishment risk more directly.

## 5. Conclusions

This retrospective national surveillance analysis across the complete 2005–2024 period (20 consecutive years) found persistent predominance of imported malaria, sparse autochthonous cases, substantial temporal change in the relative distribution of *P. vivax* and *P. falciparum*, declining API and testing-volume indicators, and geographic variation in crude 2024 reported case rates. The results are surveillance signals rather than evidence of the effectiveness of specific control interventions. Continued diagnostic capacity, prompt investigation and management of imported infections, and surveillance for possible re-establishment of local transmission remain important in Oman’s elimination setting.

## Figures and Tables

**Table 1:** National Malaria Surveillance Indicators in Oman (2005–2024).

Year	API (Per 1000)	SPR (%)	ABER (%)	Imported	Autochthonous	Total Cases	Blood Slides Examined
2005	0.217	0.21	10.32	544	0	544	258,981
2006	0.172	0.18	9.42	443	0	443	242,635
2007	0.257	0.29	8.91	701	4	705	244,346
2008	0.337	0.39	8.55	957	8	965	245,113
2009	0.283	0.38	7.40	898	0	898	234,803
2010	0.369	0.53	6.98	1169	24	1193	226,009
2011	0.435	0.57	7.60	1518	13	1531	267,353
2012	0.566	0.76	7.45	2029	22	2051	269,990
2013	0.376	0.63	5.97	1440	11	1451	230,041
2014	0.251	0.54	4.63	986	15	1001	184,996
2015	0.198	0.41	4.77	818	4	822	198,589
2016	0.183	0.40	4.61	804	3	807	203,409
2017	0.236	0.66	3.57	1059	19	1078	162,628
2018	0.200	0.58	3.47	891	30	921	159,535
2019	0.290	1.06	2.74	1323	15	1338	126,680
2020	0.060	0.64	0.94	276	0	276	43,284
2021	0.038	0.65	0.58	172	0	172	26,407
2022	0.052	0.49	1.07	257	1	259	52,630
2023	0.056	0.82	0.67	286	1	287	34,828
2024	0.064	0.95	0.67	334	4	338	35,534

Imported infections dominated the classified case series throughout 2005–2024, while autochthonous cases were sparse. Restoration of 2006–2009 added 2999 imported and 12 autochthonous cases to the analytical series. The corrected origin data also retain 1323 imported and 15 autochthonous cases in 2019; in 2022, one of the 259 reported cases was not assigned to either origin category. Across the full 20-year series, totals were 16,905 imported cases, 174 autochthonous cases, and one unclassified-origin case, yielding 17,080 cases overall.

**Table 2:** Geographic Distribution of Reported Malaria Cases and Crude Population-Referenced Case Rates, Oman, 2024.

Governorate	Reported Cases	End-2024 Registered Population	Crude Reported Case Rate per 100,000
Muscat	124	1,498,521	8.3
Al Buraymi	46	135,821	33.9
North Al Batinah	27	925,163	2.9
Dhofar	26	529,574	4.9
Ad Dakhliyah	25	560,483	4.5
South Al Batinah	22	566,771	3.9
South Ash Sharqiyah	21	369,528	5.7
North Ash Sharqiyah	21	321,462	6.5
Adh Dhahirah	16	244,373	6.5
Al Wusta	9	61,226	14.7
Musandam	1	55,150	1.8
Oman total	338	5,268,072	6.4

Note: Population denominator = registered population at end of 2024, National Centre for Statistics and Information. Rates are crude reported case rates and combine imported and locally acquired cases; they are not estimates of local transmission incidence.

**Table 3:** Temporal Relationship Between Malaria Testing Intensity and Slide Positivity Rate in Oman (20 years, 2005–2024).

Year	Blood Slides Examined	SPR (%)	ABER (%)
2005	258,981	0.21	10.32
2006	242,635	0.18	9.42
2007	244,346	0.29	8.91
2008	245,113	0.39	8.55
2009	234,803	0.38	7.40
2010	226,009	0.53	6.98
2011	267,353	0.57	7.60
2012	269,990	0.76	7.45
2013	230,041	0.63	5.97
2014	184,996	0.54	4.63
2015	198,589	0.41	4.77
2016	203,409	0.40	4.61
2017	162,628	0.66	3.57
2018	159,535	0.58	3.47
2019	126,680	1.06	2.74
2020	43,284	0.64	0.94
2021	26,407	0.65	0.58
2022	52,630	0.49	1.07
2023	34,828	0.82	0.67
2024	35,534	0.95	0.67

Regression summary (*n* = 20): B = −0.00000147 (95% CI −0.00000254 to −0.00000040), standardized β = −0.563, *p* = 0.010, R^2^ = 0.317; Newey–West lag-1 *p* = 0.019. Note: SPR = Slide Positivity Rate; ABER = Annual Blood Examination Rate. The coefficient is an ecological annual association and does not imply that changes in testing volume caused changes in SPR.

**Table 4:** Origin of Malaria Cases in Oman (20 years, 2005–2024).

Year	Imported *n* (%)	Autochthonous *n* (%)	Unclassified *n* (%)	Total
2005	544 (100.00)	0 (0.00)	0 (0.00)	544
2006	443 (100.00)	0 (0.00)	0 (0.00)	443
2007	701 (99.43)	4 (0.57)	0 (0.00)	705
2008	957 (99.17)	8 (0.83)	0 (0.00)	965
2009	898 (100.00)	0 (0.00)	0 (0.00)	898
2010	1169 (97.99)	24 (2.01)	0 (0.00)	1193
2011	1518 (99.15)	13 (0.85)	0 (0.00)	1531
2012	2029 (98.93)	22 (1.07)	0 (0.00)	2051
2013	1440 (99.24)	11 (0.76)	0 (0.00)	1451
2014	986 (98.50)	15 (1.50)	0 (0.00)	1001
2015	818 (99.51)	4 (0.49)	0 (0.00)	822
2016	804 (99.63)	3 (0.37)	0 (0.00)	807
2017	1059 (98.24)	19 (1.76)	0 (0.00)	1078
2018	891 (96.74)	30 (3.26)	0 (0.00)	921
2019	1323 (98.88)	15 (1.12)	0 (0.00)	1338
2020	276 (100.00)	0 (0.00)	0 (0.00)	276
2021	172 (100.00)	0 (0.00)	0 (0.00)	172
2022	257 (99.23)	1 (0.39)	1 (0.39)	259
2023	286 (99.65)	1 (0.35)	0 (0.00)	287
2024	334 (98.82)	4 (1.18)	0 (0.00)	338
2005–2024 Total	16,905 (98.98)	174 (1.02)	1 (0.01)	17,080

Note: Percentages use the annual total as denominator. One 2022 case was unclassified by origin and was included in the descriptive total but excluded from the imported-versus-autochthonous Poisson rate model. The full-period temporal change in the autochthonous case rate was not statistically significant; therefore, Table 4 should not be interpreted as demonstrating interruption or resurgence of local transmission.

**Table 5:** Longitudinal Distribution of *Plasmodium* Species in Oman (2005–2024).

Year	*P. vivax*	*P. falciparum*	Total	*P. vivax* (%)	*P. falciparum* (%)
2005	385	153	538	71.6	28.4
2006	341	100	441	77.3	22.7
2007	602	101	703	85.6	14.4
2008	870	94	964	90.2	9.8
2009	718	178	896	80.1	19.9
2010	1039	151	1190	87.3	12.7
2011	1422	109	1531	92.9	7.1
2012	1963	87	2050	95.8	4.2
2013	1366	85	1451	94.1	5.9
2014	865	134	999	86.6	13.4
2015	701	118	819	85.6	14.4
2016	636	169	805	79.0	21.0
2017	612	453	1065	57.5	42.5
2018	318	587	905	35.1	64.9
2019	206	1080	1286	16.0	84.0
2020	25	237	262	9.5	90.5
2021	41	128	169	24.3	75.7
2022	136	110	246	55.3	44.7
2023	195	89	284	68.7	31.3
2024	224	113	337	66.5	33.5

Rate-model note: Robust Poisson models used category, centered year, and category × year with the annual two-species classified total as offset. *P. falciparum* aRR = 1.142 (95% CI 1.068–1.221, *p* < 0.001); *P. vivax* aRR = 0.952 (95% CI 0.919–0.987, *p* = 0.007); species-by-year ratio of rate ratios = 1.199 (95% CI 1.112–1.293, *p* < 0.001).

**Table 6:** Passive and Active Malaria Testing in Oman (2005–2024).

Year	Passive Tested	Active Tested	Other/Unclassified Tested	Total Tested	Passive Tested (%)	Active Tested (%)
2005	204,009	54,972	0	258,981	78.8	21.2
2006	NR	NR	NR	242,635	NR	NR
2007	NR	NR	NR	244,346	NR	NR
2008	NR	NR	NR	245,113	NR	NR
2009	NR	NR	NR	234,803	NR	NR
2010	136,984	89,025	0	226,009	60.6	39.4
2011	138,220	129,133	0	267,353	51.7	48.3
2012	129,792	140,198	0	269,990	48.1	51.9
2013	113,154	116,887	0	230,041	49.2	50.8
2014	80,675	87,368	16,953	184,996	43.6	47.2
2015	88,077	110,512	0	198,589	44.4	55.6
2016	87,157	116,252	0	203,409	42.8	57.2
2017	69,435	93,193	0	162,628	42.7	57.3
2018	71,507	88,028	0	159,535	44.8	55.2
2019	55,359	71,321	0	126,680	43.7	56.3
2020	28,182	15,102	0	43,284	65.1	34.9
2021	25,126	1281	0	26,407	95.1	4.9
2022	45,794	6836	0	52,630	87.0	13.0
2023	32,265	2558	5	34,828	92.6	7.3
2024	24,485	10,800	249	35,534	68.9	30.4

Note: Values are numbers of slides tested through the respective surveillance approaches. NR = not reported in the compiled approach-specific series; 2006–2009 remain included in the 20-year analyses of national indicators, case origin, and species. Other/unclassified testing is shown separately where reported. Confirmed malaria-positive cases by detection approach were unavailable, so no inferential comparison of surveillance effectiveness was undertaken.

**Table 7:** Linear Regression Analysis of Malaria Indicator Trends (2005–2024).

Indicator	B	SE	β	t	*p*	R^2^	Trend Direction
API	−0.0142	0.0045	−0.597	−3.158	0.005	0.357	decreasing
SPR	0.0283	0.0062	0.732	4.561	<0.001	0.536	increasing
ABER	−0.5373	0.0221	−0.985	−24.326	<0.001	0.970	decreasing
Blood slides examined	−13,425	1468	−0.907	−9.143	<0.001	0.823	decreasing

Note: B = unstandardized annual slope; SE = standard error; β = standardized coefficient; R^2^ = coefficient of determination. Calendar year was entered as a continuous predictor. Each model used all 20 years from 2005 through 2024. Ordinary least-squares estimates are shown; Newey–West lag-1 sensitivity analyses did not change the inferential conclusions.

**Table 8:** Joinpoint Sensitivity Analysis of National Malaria Indicators, Oman, 2005–2024.

Indicator	Selected Joinpoint (s)	Segment	APC (%)	95% CI	*p*
API	2012	2005–2012	13.74	3.44 to 25.08	0.011
API	2012	2012–2024	−17.96	−22.06 to −13.64	<0.001
SPR	2010	2005–2010	22.98	12.10 to 34.92	<0.001
SPR	2010	2010–2024	2.62	−0.26 to 5.58	0.072
ABER	2018, 2021	2005–2018	−7.74	−9.82 to −5.61	<0.001
ABER	2018, 2021	2018–2021	−43.28	−49.18 to −36.70	<0.001
ABER	2018, 2021	2021–2024	2.27	−11.60 to 18.31	0.748
Blood slides examined	2018, 2021	2005–2018	−3.06	−5.35 to −0.71	0.014
Blood slides examined	2018, 2021	2018–2021	−44.21	−50.28 to −37.41	<0.001
Blood slides examined	2018, 2021	2021–2024	8.09	−7.22 to 25.93	0.296

Note: APC = Annual Percent Change; API = Annual Parasite Incidence; SPR = Slide Positivity Rate; ABER = Annual Blood Examination Rate. Models were fitted on the natural-log scale with a maximum of two joinpoints, at least three annual observations per segment, and sequential residual-permutation testing (1999 permutations). Joinpoint analysis was not applied to sparse autochthonous case counts.

## Data Availability

No new primary data were generated. The study analysed aggregated, publicly available malaria surveillance information reported by the Oman Ministry of Health and population statistics published by the Oman National Centre for Statistics and Information. The annual report series is available through the Ministry of Health statistics portal; availability of individual-level or additional disaggregated surveillance data remains subject to the original data custodians.

## References

[1] World Health Organization World Malaria Report 2025. https://www.who.int/teams/global-malaria-programme/reports/world-malaria-report-2025.

[2] Lu G., Zhang D., Chen J., Cao Y., Chai L., Liu K., Chong Z., Zhang Y., Lu Y., Heuschen A. (2023). Predicting the risk of malaria re-introduction in countries certified malaria-free: A systematic review. Malar. J..

[3] Lusiyana N., Ahmad R.A., Elyazar I., Murhandarwati E.E.H. (2025). Imported malaria: A silent and forgotten threat in malaria-free zones?. Malar. J..

[4] Jiram A., Jelip J., Noordin R., Musa K., Azian Yusuf N., Amir N., Rasyidina A., Ismail N., Hussin Z., Ismail A. (2025). Persistent low-density infection in areas at risk of malaria reintroduction in Malaysia. PLoS ONE.

[5] World Health Organization World Malaria Report 2010. https://www.who.int/publications/i/item/9789241564106.

[6] Al Mukhaini S.K., Mohammed O.A., Gerbers S., Al Awaidy S.T. (2023). The progress towards national malaria elimination: The experience of Oman. Oman Med. J..

[7] Snow R.W., Amratia P., Zamani G., Mundia C.W., Noor A.M., Memish Z.A., Zahrani M., Al Jasari A., Fikri M., Atta H. (2013). The malaria transition on the Arabian Peninsula: Progress toward a malaria-free region between 1960–2010. Adv. Parasitol..

[8] Singh N., Bharti P.K., Kumre N.S. (2016). Active v. passive surveillance for malaria in remote tribal belt of Central India: Implications for malaria elimination. Pathog. Glob. Health.

[9] Simon B., Sow F., Al Mukhaini S.K., Al-Abri S., Ali O.A., Bonnot G., Bienvenu A., Petersen E., Picot S. (2017). An outbreak of locally acquired *Plasmodium vivax* malaria among migrant workers in Oman. Parasite.

[10] World Health Organization, Regional Office for the Eastern Mediterranean (2012). Report on the Regional Workshop on Strengthening Quality Management Systems for Parasitological Diagnosis of Malaria, Muscat, Oman, 17–21 September 2011.

[11] Kaur J., Baharia R., Dattani M. (2024). Assessment of the quality of malaria surveillance and laboratory services for diagnosis in three districts of Gujarat state, India. Front. Public Health.

[12] Al-Awadhi M., Ahmad S., Iqbal J. (2021). Current status and the epidemiology of malaria in the Middle East region and beyond. Microorganisms.

[13] Alharbi B.F., Ahmed M.A. (2026). Malaria in the 21st century: Global disease burden, epidemiological insights, and strategic control approaches. Biology.

[14] Feachem R.G.A., Phillips A.A., Hwang J., Cotter C., Wielgosz B., Greenwood B.M., Sabot O., Rodriguez M.H., Abeyasinghe R.R., Ghebreyesus T.A. (2010). Shrinking the malaria map: Progress and prospects. Lancet.

[15] Directorate General of Planning and Studies, Ministry of Health, Sultanate of Oman Annual Health Reports, 2005–2024. https://www.moh.gov.om/en/statistics/annual-health-reports/.

[16] World Health Organization (2012). Disease Surveillance for Malaria Elimination: An Operational Manual.

[17] National Centre for Statistics and Information, Sultanate of Oman (2025). Statistical Year Book 2025: Population Registered by Governorate at the End of 2024.

[18] Hadi A.H.A., Sharwani A.O.M., Madkhali A.M.A., Khamjan A.A.N., Ghazwi A.M.A., Ahmed A.B.M.Q. (2025). Epidemiological profile and current trends of malaria in the Middle East: An updated review. Saudi J. Med. Public Health.

[19] World Health Organization (2024). World Malaria Report 2024.

[20] Alharbi B.F., Ahmed M.A. (2026). Malaria in travelers and local populations: Comprehensive study of incidence patterns and origin-based classification in Saudi Arabia. PLoS ONE.

[21] Cao Y., Wu H., Zhang Y., Wu X., Li J., Chen H., Gao W. (2025). Time trends in malaria incidence from 1992 to 2021 in high-risk regions: An age-period-cohort analysis based on the Global Burden of Disease Study 2021. Int. J. Infect. Dis..

[22] Tseroni M., Georgitsou M., Baka A., Pinaka O., Pervanidou D., Tsironi M., Bleta P., Charvalakou M., Psinaki I., Dionysopoulou M. (2020). The importance of an active case detection (ACD) programme for malaria among migrants from malaria-endemic countries: The Greek experience in a receptive and vulnerable area. Int. J. Environ. Res. Public Health.

